# Isolation of the Binding Protein of Periplocoside E from BBMVs in Midgut of the Oriental Amyworm *Mythimna separata* Walker (Lepidoptera: Noctuidae) through Affinity Chromatography

**DOI:** 10.3390/toxins8050139

**Published:** 2016-05-04

**Authors:** Mingxing Feng, Zhenyu He, Yuanyuan Wang, Xiufang Yan, Jiwen Zhang, Zhaonong Hu, Wenjun Wu

**Affiliations:** 1 Key Laboratory of Botanical Pesticide R & D in Shaanxi Province, Yangling 712100, Shaanxi, China; fengmx2010@126.com (M.F.); hezhenyubobo@163.com (Z.H.); nwzjw@nwsuaf.edu.cn (J.Z.); wuwenjun@nwsuaf.edu.cn (W.W.); 2 Institute of Pesticide Science, College of Plant Protection, Northwest A & F University, Yangling 712100, Shaanxi, China; yuanquanerm@163.com (Y.W.); xiufangyan@163.com (X.Y.)

**Keywords:** periplocosides E, affinity chromatography, binding protein, *Mythimna separata*

## Abstract

Periplocosides, which are insecticidal compounds isolated from the root bark of *Periploca sepium* Bunge, can affect the digestive system of insects. However, the mechanism though which periplocosides induces a series of symptoms remains unknown. In this study, affinity chromatography was conducted by coupling periplocoside E-semi-succinic acid ester with epoxy amino hexyl (EAH) sepharose 4B. Sodium dodecyl sulfonate-polyacrylamide gelelectrophoresis (SDS-PAGE) was performed to analyze the fraction eluted by periplocoside E. Eight binding proteins (luciferin 4-monooxygenase, aminopeptidase N, aminopeptidase N3, nicotinamide adenine dinucleotide health (NADH) dehydrogenase subunit 5, phosphatidylinositol 3-phosphate 3-phosphatase myotubularin, actin, uncharacterized family 31 glucosidase KIAA1161, and 2OG-Fe(2) oxygenase superfamily protein) were obtained and identified through liquid chromatography/quadrupole-time of flight-mass spectrometry (LC/Q-TOF-MS) analysis of the midgut epithelium cells of *Mythimna separata* larvae. Aminopeptidase N and N3 are potential putative targets of periplocosides. This study establishes the foundation for further research on the mechanism of action and target localization of periplocosides in agricultural pests.

## 1. Introduction

Global pesticide production should be highly effective, confer low toxicity and high safety coefficient, and exhibit satisfactory selectivity and environmental compatibility. Botanical pesticides have been widely investigated and applied because of their desirable properties [1]. New natural biological active compounds must also be discovered to promote the development of environmentally friendly pesticides [2]. As such, the use of biological active ingredients [3] or action targets [4] as guides is an important aspect in research and development of botanical pesticides.

*Periploca sepium* Bunge, a traditional Chinese herbal medicinal plant belonging to the Asclepiadaceae family, exhibits medicinal and insecticidal properties [5,6]. *P. sepium* secondary metabolites contain periplocosides, which are pregnane glycosides with evident insecticidal activity against several insect species [7]. These compounds possess a special mode of action that involves obvious swelling of the abdomen, and, thus far, no other modes of insecticidal action have been recorded [8]. Fluorescence localization analysis indicated that periplocoside NW (PSNW) can bind to the midgut cells of *Mythimna separata* larvae [9]. Ultrastructural observations showed that microvilli, organelle, and cytomembrane are destructed in the midgut cells of *M. separata* larvae [10]. Research on histopathological effects indicated that brush-border membrane vesicles (BBMVs) from the midgut epithelium cells of *M. separata* larvae are the initial action site; one or more binding sites may also exist in the larvae [11]. However, the specific binding sites and insecticidal mechanism of periplocosides in *M. separata* larval midgut cells remain unclear.

The cellular targets of bioactive compounds can be identified using several approaches. Affinity purification is a classic and commonly used technique to identify receptors of insecticidal active substances [12]. In contrast to other approaches, affinity-based approaches are employed to identify directly small-molecule binding proteins.

**Figure 1:**
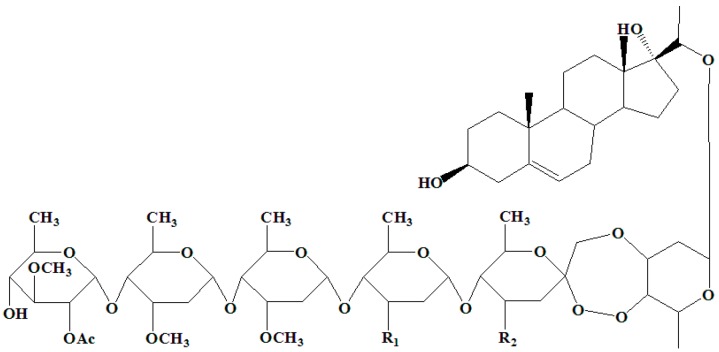
Structure of periplocoside E, P, and T. Periplocoside E: R_1_ = OCH_3_, R_2_ = OCH_3_. Periplocoside P: R_1_ = OH, R_2_ = OCH_3_. Periplocoside T: R_1_ = OH, R_2_ = OH.

In this research, although periplocoside E has relatively low insecticidal activity or is inactive compared with periplocoside P and T [13], periplocoside E was selected based on the structure and high consumption of experimental materials to determine the binding protein of periplocosides compounds. The chemical structure of periplocoside E is similar to those of periplocoside P and T in terms of the common features except for one or two substituents (Figure 1). Thus, we separated the binding protein of periplocoside E from the BBMVs from the midgut epithelium of *M. separata* larvae through affinity chromatography to elucidate the mode of action of the compound.

## 2. Results and Discussion

### 2.1. Identification of the Ligand (Periplocoside E-Semi-Succinic Acid Ester)

**Figure 2:**
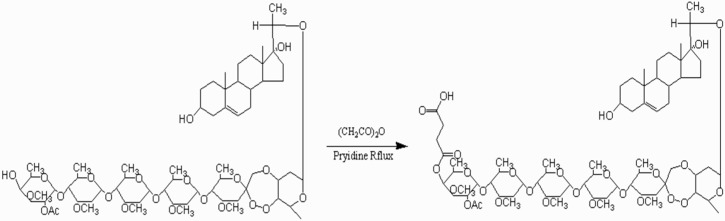
Synthetic route of periplocoside E-semi-succinic acid ester.

The ligand, periplocoside E-semi-succinic acid ester, was synthesized (Figure 2) and identified by electrospray ionization-mass spectrometry (ESI-MS) and nuclear magnetic resonance (NMR) analysis.

**Figure 3:**
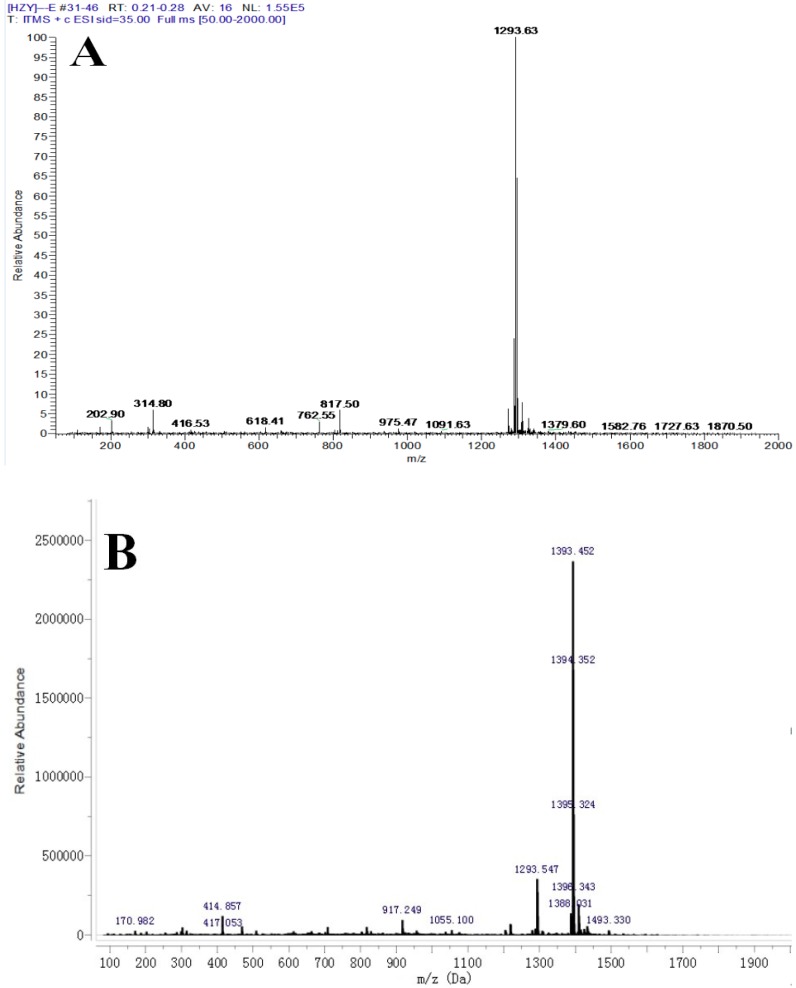
Mass spectrum (MS) spectrum of periplocoside E (**A**) and periplocoside E-semi-succinic acid ester (**B**).

**Figure 4:**
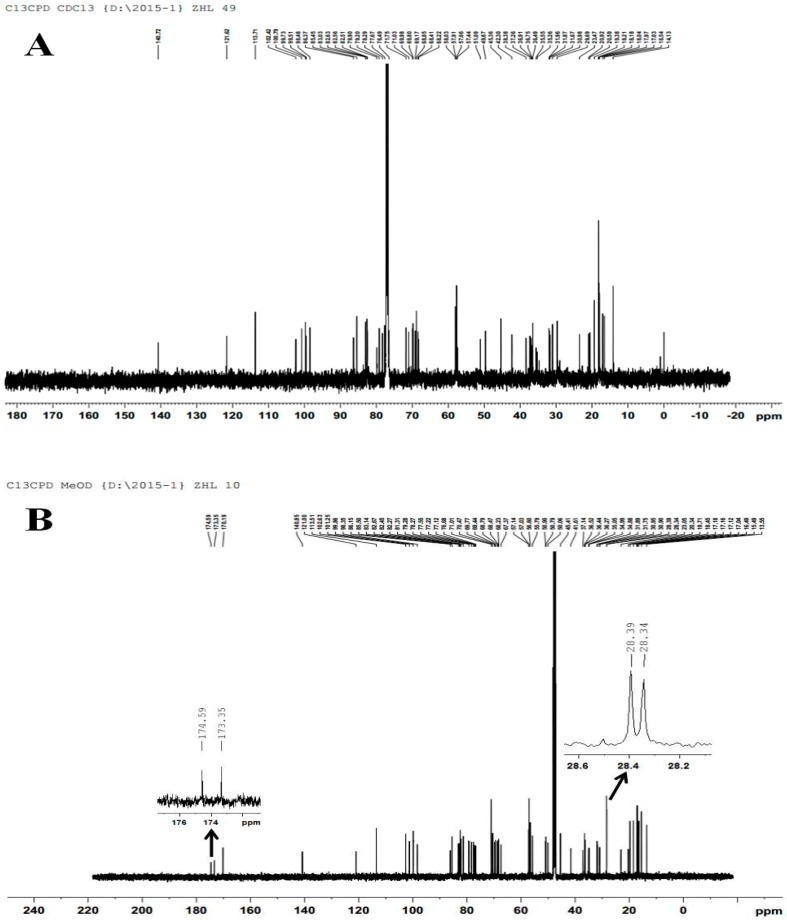
Nuclear magnetic resonance (NMR) spectra of periplocoside E (**A**) and periplocoside E-semi-succinic acid ester (**B**).

According to ESI-MS identification, the quasi-molecular ion peaks [M + Na] + of periplocoside E was located at *m*/*z* 1293.48 (Figure 3A), and the quasi-molecular ion peak [M + Na] + of periplocoside E-semi-succinic acid ester was found at *m*/*z* 1393.56 (Figure 3B). The ESI-MS value of periplocoside E-semi-succinic acid ester is consistent with the theoretical value. Further analysis by NMR identification showed that periplocoside E-semi-succinic acid ester (Figure 4B) contains four more lines than that of periplcoside E (Figure 4A). The NMR spectra of these lines are δC 174.59, 173.35, 28.39, and 28.34, respectively. Among these lines, δC 174.59 and 173.35 represent two carbonyl carbon atoms in the succinic anhydride structure, and δC 28.39 and 28.34 are assigned to two methylene carbon atoms. Hence, periplocoside E-semi-succinic acid ester was successfully synthesized.

### 2.2. Isolation of Binding Proteins

**Figure 5:**
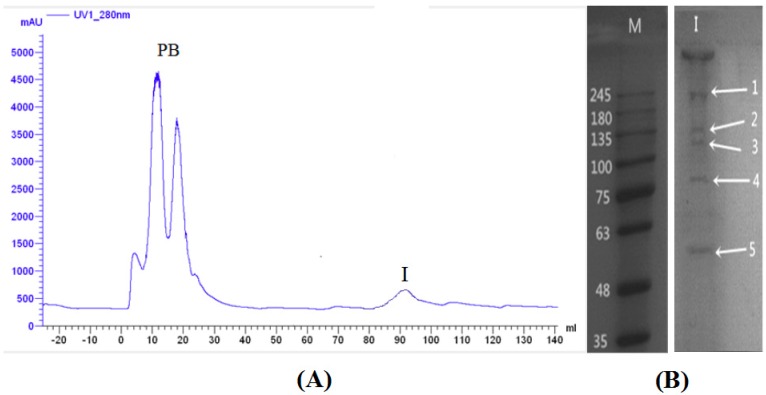
Affinity chromatography using periplocoside E-Sepharose of brush-border membrane vesicle (BBMV) proteins extracted from *M. separata* larvae. (**A**) Phosphate buffer (PB) is the unbound protein eluted with a binding buffer; I is eluted with periplocoside E dissolved in the binding buffer; (**B**) 12% sodium dodecyl sulfonate-polyacrylamide gelelectrophoresis (SDS-PAGE) of fractions; M: protein marker.

Our preliminary results revealed that the BBMVs from the midgut epithelium cells of *M. separata* larvae possess the initial action site of periplocosides [11]. Therefore, the BBMVs of *M. separata* larvae was isolated and subjected to affinity chromatography to separate the binding proteins of periplocosides. Two fractions, namely, Fractions 1 (Phosphate buffer (PB), unbound protein eluted with a binding buffer) and 2 (I, eluted with periplocoside E dissolved in the binding buffer) were collected (Figure 5A).

SDS-PAGE (Figure 5B) and LC/Q-TOF-MS analysis was conducted to resolve and evaluate proteins eluted by excess periplocoside E. Table 1 shows the binding proteins obtained from the NCBI database.

Table 1 shows that eight binding proteins of periplocoside E (luciferin 4-monooxygenase, aminopeptidase N, aminopeptidase N3, NADH dehydrogenase subunit 5, phosphatidylinositol 3-phosphate 3-phosphatase myotubularin, actin, uncharacterized family 31 glucosidase KIAA1161, and 2OG-Fe(2) oxygenase superfamily protein) were obtained from the *M. separata* midgut through affinity chromatography. Based on previous observations of symptoms, a preliminary research mechanism, the function of the obtained proteins, and the determination results of enzyme activity (data not published), we speculate that aminopeptidase N and aminopeptidase N3 are likely to be the putative target of periplocosides.

Symptoms of insect poisoning by periplocosides are characterized by a cessation of feeding and apparent abdomen swelling [8]. Further ultrastructural observation showed that microvilli on midgut epithelial cells are severely damaged [10]. We hypothesized that periplocosides may disrupt microvilli on the epithelial cells of the midgut of insects by interacting with proteins on the microvillar membrane or those associated with the membrane; these interactions may affect the normal functions of insects. Similar to periplocosides, the Bt toxin [14] and celangulins [15] act on the midgut of insects. Thus, periplocosides may have the same target protein as that of the Bt toxin and celangulins.

Research on the Bt toxin indicated that aminopeptidase N (APN) [16], cadherin-like protein[17], alkaline phosphatase (ALP) [18], and actin [19] are the receptors of the detoxification function. V-ATPase is a potential putative target protein of celangulins [20]. Through comparison with the receptors of the Bt toxin and celangulins, we found actin and aminopeptidase N (APN) in the present research. Actin is an abundant protein in eukaryotic cells [21]. Actin’s main function is to form microfilaments to maintain cell morphology; actin also exhibits other multiple functions, such as cell motility, cell division, and organelle movement [22,23]. Many of these processes are mediated by extensive and intimate interactions of actin and cellular membranes [24]. However, in terms of selectivity, actin has less possibility of becoming a target protein of pesticides because of its highly conservative nature in insect cells. Further studies on actin must be performed to exclude and validate this conjecture.

Aminopeptidase-N (APN) is an exopeptidase that is abundant in the BBMVs of many lepidopteran and dipteran insects. APN catalyzes the removal of N-terminal neutral amino acids [25]. APN is a common component of lepidoptera larval midgut and is used as a marker enzyme to assess the purity of BBMVs from insect larval midguts [26]. APN belongs to the family of zinc-dependent enzyme peptide with the zinc finger structure and *N*-glycosylation sites (Asn-X-Ser/Thr). The phosphatidyl inositol signal sequence is located in the *C*-terminal, and the signal peptide sequence is situated in the *N*-terminal. APN is connected to BBMVs in the insect midgut through the glycosylated phosphatidylinositol [27]. 

The most studied and reported aspect of APN is the Bt toxin. APN is the first toxin receptor protein that plays a role in the mechanism of the Bt toxin [16]. Knight *et al.* [28] and Gill *et al.* [29] cloned a 120-kD receptor-related protein gene combined with the Cry1Ac of *Manduca sexta* and *Heliothis virescen*; these studies applied the method of cDNA library and showed that APN is the encoding protein. Moreover, binding experiments showed that APN is the receptor of *Lymantria dispar*, *Plutella xylostella*, and *Trichoplusia ni* combined with Cry1Ac [30,31]. Gill *et al.* [32] indicated that the APN of *Manduca sexta* in the midguts of *Drosophila melanogaster* larvae is not sensitive to Cry1Ac; they found that this structure led to the presence of *D. melanogaster* larvae, which is sensitive to Cry1Ac. Furthermore, multiple aminopeptidase-N protein sequence alignments showed that the first 40 residues of human, rat, pig, and mouse enzymes are characterized by signal sequence, which may function as a membrane anchor [33,34]. Some reports showed that human and pig coronaviruses also use aminopeptidase N as receptor in their target tissues [35,36]. APN has a long history of existence as a target receptor.

In conclusion, the eight proteins isolated through affinity chromatography are potential binding proteins that interact with periplocosides. Aminopeptidase-N may be the target protein of periplocosides because it induces a series of symptoms to destroy the digestive system. However, further in-depth studies are needed to qualify and validate whether APN is the receptor of periplocosides. The presence of periplocosides in the midgut of insects, which leads to the death of the insect, should be evaluated using the interaction of periplocosides with APN. This work will serve as an important guide in elucidating the mechanism of periplocosides.

## 3. Conclusions

Eight potential binding proteins of periplocoside E were isolated from the midgut BBMVs of *M. separata* larvae through affinity chromatography. These proteins include luciferin 4-monooxygenase, aminopeptidase N, aminopeptidase N3, NADH dehydrogenase subunit 5, phosphatidylinositol 3-phosphate 3-phosphatase myotubularin, actin, uncharacterized family 31 glucosidase KIAA1161, and 2OG-Fe(2) oxygenase superfamily protein. The functions of some of the important proteins linked to the symptoms caused by periplocosides were also analyzed. We speculate that aminopeptidase N (APN) is the putative target protein of periplocosides. However, further studies are needed to verify the accuracy of this assumption.

## 4. Materials and Methods

### 4.1. Insects

The fifth instar larvae of *M. separata* were provided by the Institute of Pesticide Science, Northwest A & F University (NWAFU) (Yangling, China). A colony of *M. separata* was cultured in the laboratory for 20 years at 25 °C, 70% relative humidity, and a photoperiod of 16 h of light, with 8 h of darkness under periodic introduction of field-collected insects.

### 4.2. Chemicals

Periplocoside E was extracted and purified from *P. sepium* Bunge (root barks) in the Institute of Pesticide Science, Northwest A & F University (NWAFU, Yangling, China) [37]. The chemical structure of PS-E is shown in Figure 1. PS-E has a purity of over 98% based on high performance liquid chromatography (HPLC) analysis. EAH sepharose 4B was purchased from general electronics (GE) Healthcare (Beijing, China). Tris, NaCl, HCl, acetic acid, sodium acetate, and all other chemicals were purchased from Solarbio (Beijing, China).

### 4.3. Preparation of Brush Border Membrane Vesicles (BBMV)

The fifth instar larvae of *M. separata* Walker were starved for 12 h before the experiment. The midguts were then dissected. Peritrophic matrix and gut contents were removed. The remaining tissues were washed in an ice-cold 0.7% NaCl solution. The cleaned tissue was dried on filter paper, weighed, and used to separate the BBMVs according to the differential magnesium precipitation method with some modifications [38]. The final BBMV pellet was dissolved in an ice-cold dissolution buffer (150 mM NaCl, 5 mM EGTA, 1 mM PMSF, 20 mM Tris-HCl, and 1% CHAPS). Protein concentration was quantified by Bradford Assay, and the prepared BBMVs were stored at −80 °C until analysis.

### 4.4. Synthesis of Ligand

The ligand, periplocoside E-semi-succinic acid ester, was synthesized using periplocoside E and succinic anhydride (Figure 2).

Briefly, 100 mg of periplocoside E (0.08 mmol) and 100 mg of succinic anhydride (1 mmol) were placed in a reaction vessel and dissolved in 20 mL of anhydrous pyridine. The mixture was reacted at 120 °C through a heating reflux method. Subsequently, 20 mL of methanol was added to terminate the completed reaction (surveillance by thin layer chromatography (TLC)). The solvent was evaporated at 60 °C by using rotary evaporators (Thermo Fisher, Beijing, China). The remaining dry powder was dissolved in methyl alcohol and further separated by column chromatography. The structure was characterized by MS and ^13^C-NMR and MS analyses.

### 4.5. Preparation of the Medium and Coupling of the Ligand

The medium, namely, epoxy amino hexyl (EAH)-activated sepharose 4B, was prepared according to the operation manual. The lyophilized powder of EAH Sepharose 4B (2.5 g) was sufficiently swelled in 100 mL of 20% ethanol. When the medium swelled, the ethanol solution was decanted, and the required amount of the matrix was washed five times on a glass filter (porosity G3) with distilled water (pH 4.5). The matrix was subjected to sedimentation using 80 mL of 0.5 mol/L NaCl.

The carbodiimide method was used for ligand coupling. Periplocoside E-semi-succinic acid ester (ligand) was dissolved in the coupling solution (50% ethylene glycol solution) and added to the matrix (matrix:ligand = 1:1) to form a suspension. Briefly, 0.1 mol/L carbodiimide was added to the suspension. The superfluous medium was eluted with three cycles of an alternating pH elution buffer (buffer 1: 0.1 mol/L acetic acid/sodium acetate containing 0.5 mol/L NaCl, pH 4.0; buffer 2: 0.1 mol/L Tris-HCl containing 0.5 mol/L NaCl, pH 8.0). The volumes of each elution buffer were at least five times the medium volumes.

### 4.6. Affinity Chromatography

The prepared EAH sepharose 4B was packed to the column of an AKTA protein purification system (GE Healthcare, Beijing, China). The packing flow rate was maintained for three bed volumes after reaching a constant bed height was reached.

After loading the BBMVs of the *M. separata*, the medium was washed with the binding buffer (0.1 mol/L NaH_2_PO_4_/Na_2_HPO_4_, pH 7.4 containing 0.5 mol/L NaCl) until the base line become stable. For competitive elution, periplocoside E was dissolved in the binding buffer. The elution peak was collected by washing the medium with the elution buffer. The affinity medium was then washed with an alternating high pH (0.1 mol/L Tris-HCl, pH 8.0 containing 0.5 mol/L NaCl) and low pH (0.1 mol/L acetic acid/ sodium acetate, pH4.0 containing 0.5 mol/L NaCl) buffer solution to regenerate the medium. This cycle was repeated three times.

### 4.7. SDS-PAGE and LC/Q-TOF-MS Analysis

The collected binding protein solution was dialyzed to decrease its high salt concentration. The samples were then lyophilized. SDS-PAGE (12% separating gel and 5% spacer gel) was conducted by dissolving the sample in the Tris-HCl (pH 7.2). The whole visible lane was obtained for protein MS identification. Protein MS was implemented in Beijing Protein Institute (Beijing, China) by using LC/Q-TOF-MS.

## Figures and Tables

**Table 1:** Binding proteins of periplocoside E from *M. separata* midgut recognized by affinity chromatography.

Number	Protein ID	Protein Mass	Protein
1	gi|512918093	63095	Luciferin 4-monooxygenase
2	gi|15212557	114719	Aminopeptidase N
3	gi|21327773	107153	Aminopeptidase N3
4	gi|253807662	66764	Nicotinamide adenine dinucleotide health (NADH) dehydrogenase subunit 5
5	gi|520836887	78123	Phosphatidylinositol 3-phosphate 3-phosphatase myotubularin
6	gi|323435326	31100	Actin
7	gi|512901127	73062	Uncharacterized family 31 glucosidase KIAA1161
8	gi|399942986	86025	2OG-Fe(2) oxygenase superfamily protein
